# Assessment of *Vibrionaceae* prevalence in seafood from Qidong market and analysis of *Vibrio parahaemolyticus* strains

**DOI:** 10.1371/journal.pone.0309304

**Published:** 2024-08-22

**Authors:** Qinglian Huang, Yiquan Zhang, Miaomiao Zhang, Xue Li, Qinjun Wang, Xianyi Ji, Rongrong Chen, Xi Luo, Shenjie Ji, Renfei Lu

**Affiliations:** 1 School of Medicine, Nantong University, Nantong, Jiangsu, China; 2 Department of Clinical Laboratory, Nantong Third People’s Hospital, Affiliated Nantong Hospital 3 of Nantong University, Nantong, Jiangsu, China; 3 Department of Clinical Laboratory, Qidong People’s Hospital, Qidong, Jiangsu, China; University of Messina, ITALY

## Abstract

The aim of this study was to investigate the prevalence of *Vibrionaceae* family in retail seafood products available in the Qidong market during the summer of 2023 and to characterize *Vibrio parahaemolyticus* isolates, given that this bacterium is the leading cause of seafood-associated food poisoning. We successfully isolated a total of 240 *Vibrionaceae* strains from a pool of 718 seafood samples. The breakdown of the isolates included 146 *Photobacterium damselae*, 59 *V. parahaemolyticus*, 18 *V. campbellii*, and 11 *V. alginolyticus*. Among these, *P*. *damselae* and *V. parahaemolyticus* were the predominant species, with respective prevalence rates of 20.3% and 8.2%. Interestingly, all 59 isolates of *V. parahaemolyticus* were identified as non-pathogenic. They demonstrated proficiency in swimming and swarming motility and were capable of forming biofilms across a range of temperatures. In terms of antibiotic resistance, the *V. parahaemolyticus* isolates showed high resistance to ampicillin, intermediate resistance to cefuroxime and cefazolin, and were sensitive to the other antibiotics evaluated. The findings of this study may offer valuable insights and theoretical support for enhancing seafood safety measures in Qidong City.

## Introduction

The family *Vibrionaceae*, a member of the sub-phylum Gammaproteobacteria, includes genera such as *Enterovibrio*, *Grimontia*, *Photobacterium*, and *Vibrio* [1]. They are cosmopolitan, being distributed widely in marine environments, including seawater, sediments, and among marine flora and fauna [1]. The *Vibrio* genus encompasses over 100 species, with approximately 12 of these species known to be pathogenic to humans. The most prevalent among these are *V. cholerae*, *V. parahaemolyticus* and *V. vulnificus* [2, 3]. Typically, human infections from *Vibrio* species arise from the consumption of contaminated water or the ingestion of raw and undercooked contaminated seafood [2]. The number of *Vibrio* cells increases with the rise of seawater temperature, and the threat of these bacteria pose to human health seems to be on the rise with global warming [2, 4].

*V. parahaemolyticus* stands out as one of the most important pathogenic *Vibrio* species and is recognized as the leading cause of seafood-associated food poisoning [2]. The virulence of this bacterium is often attributed to the expression of thermostable direct hemolysin (TDH; *tdh*) and/or TDH-related hemolysin (TRH; *trh*) by virulent strains [5]. Additional factors such as type III secretion systems (T3SS1: VP1656-1702; T3SS2: VPA1320-1370), type VI secretion systems (T6SS1: VP1386-1420; T6SS2: VPA1024-1046), and urease (*ure*) are also implicated in the pathogenesis of *V. parahaemolyticus* [6, 7]. The *tdh* genes and the T3SS2 gene cluster are situated on a pathogenicity island termed as Vp-PAI (VPA1312-1398) [6]. Furthermore, *V. parahaemolyticus* exhibits over 70 serotypes based on somatic (O) and capsular (K) antigens. However, the ‘pandemic group’, characterized by the new O3:K6 serotype and its serovariants, has been responsible for the majority of clinical cases since 1996 [8]. Identification of *V. parahaemolyticus* isolates is typically achieved by targeting the species-specific *tlh* and *toxR* genes [9]. In contrast, isolates of the ‘pandemic group’ are confirmed through the detection of specific genetic markers including *toxRS*/*new* [10], ORF8 [11], the insertion sequence in HU-α [12], the group specific (PGS) sequence [13], and VP2905 [14].

In China, a variety of antimicrobial agents, including tetracycline, ampicillin, sulfonamides, and gentamicin, are permitted for managing bacterial infections in aquaculture. However, the improper use of antibiotics has resulted in bacteria developing resistance to multiple antibiotics. Studies have reported that certain *Vibrio* species, including *V. parahaemolyticus*, exhibit resistance to a range of antibiotics [15]. Genes that confer resistance to multiple antibiotics such as ampicillin, ceftazidime, gentamicin, chloramphenicol, and kanamycin have been identified within the genomes of *V. parahaemolyticus* isolates [16, 17]. Moreover, the ability of nearly all *V. parahaemolyticus* isolates to form robust biofilms on the surfaces of seafood products is noteworthy [9, 18]. Biofilms, which are communities of microorganisms encased in an extracellular matrix, provide the bacteria within them with enhanced resilience to harsh conditions [18, 19]. The formation of these biofilms is facilitated by various structures, including flagella, pili, adhesion proteins, and exopolysaccharides [19].

The Lvsi Fishing Ground, one of the China’s four major fishing grounds, covers an area of approximately 30,000 square kilometers and is located in Qidong, Nantong, Jiangsu Province, bordering the Yellow Sea. This region is a vital hub for marine life, offering a rich feeding and breeding environment for a variety of fish, shrimp, shellfish, crab, and other economically significant species. The presence and concentration of pathogenic *Vibrionaceae* in seafood are crucial factors impacting food safety. While numerous studies have examined the genetic, pathogenic, and phenotypic traits of significant pathogenic strains of the *Vibrionaceae* isolated in Jiangsu Province, they have predominantly concentrated on individual species, particularly *V. parahaemolyticus* [9, 20–22]. These studies have not, however, explored the prevalence of strains within the entire *Vibrionaceae* family in seafood. In this study, our aim was to assess the incidence of *Vibrionaceae* in retail seafood products available in the Qidong market during the summer of 2023. We focused on the characterization of virulence genes, biofilm formation capabilities, flagella-driven motility, and antimicrobial resistance among *V. parahaemolyticus* isolates obtained from these seafood samples.

## Materials and methods

### Sample collection

In the routine process of transporting fish to various markets, it’s common practice to utilize liquid nitrogen for rapid freezing or ice blocks for cooling to preserve the freshness and quality of the fish. Fresh seafood was collected from the seafood stalls at the retail market in Huilong Town, Qidong City, from June to November of 2023. This collection period was chosen because there is a linear correlation observed between the water temperature and the population density of *Vibrionaceae* family members [23, 24]. A total of 718 samples were collected in this study, including 30 commonly consumed seafood products. These encompassed 16 species of fish, 7 species of shrimp, 3 species of shellfish, 2 species of crab, and 2 species of cephalopods (Table 1). Most of the fish samples were collected from the viscera, gills, or heads. Tissue samples from the same individuals were placed in a sterile sampling bag. Some smaller fish were placed directly in the bag and sent to Department of Clinical Laboratory of Nantong Third People’s Hospital. Shellfish were individually bagged and, upon arrival at the laboratory, their shells were brushed and opened to extract all the contents. Shrimp were transported to the laboratory with only their heads taken for analysis, while crabs were either taken with their gills or with their detached legs. All samples must arrive at the laboratory within two hours and should be processed within half an hour of arrival.

**Table 1:** Information of seafood samples and *Vibrionaceae* family isolated from June to November of 2023.

Sample	Scientific name	Sample number	Number of isolates							
			*V. parahaemolyticus*	*P*. *damselae*	*V. mimicus*	*V. fluvialis*	*V. alginolyticus*	*V. campbellii*	*V. vulnificus*	*V. harveyi*
**Fish**	*Pampus argenteus*	67	12	6	1	0	0	2	0	0
	*Trichiurus lepturus*	79	5	17	0	0	1	2	0	0
	*Larimichthys crocea*	102	1	28	0	0	1	6	0	0
	*Dasyatis akajei*	4	1	0	0	0	1	0	0	0
	*Nibea albiflora*	1	0	1	0	0	0	0	0	0
	*Coilia nasus*	13	2	1	0	0	1	0	0	0
	*Lateolabrax japonicus*	1	0	0	0	0	0	0	0	0
	*Engraulis japonicus*	33	3	7	0	0	0	0	0	0
	*Collichthys lucidus*	102	9	11	0	0	1	2	0	0
	*Scomber japonicus*	27	0	1	0	0	0	0	0	0
	*Pennahia argentata*	2	0	0	0	0	1	0	0	0
	*Cynoglossus semilaevis*	10	0	2	0	0	0	0	0	0
	*Conger myriaster*	4	0	2	0	0	0	0	0	0
	*Ilisha elongata*	61	6	18	0	0	0	1	0	0
	*Sillago sihama*	1	1	0	0	0	0	0	0	0
	*Scomberomorus niphonius*	2	0	0	0	0	0	0	0	0
Shrimp	*Parapenaeopsis hardwickii*	12	0	5	0	1	0	1	0	0
	*Trachypenaeus curvirostris*	2	1	0	0	0	0	0	0	0
	*Penaeus japonicu*	38	6	10	0	0	0	1	0	0
	*Fenneropenaeus chinensis*	65	3	18	0	0	0	2	0	1
	*Exopalaemon carinicauda*	3	0	0	0	0	0	0	0	0
	*Oratosquilla oratoria*	1	0	0	0	0	0	0	0	0
	*Palaemon gravieri*	13	0	7	0	0	0	0	0	0
Shellfish	*Meretrix meretrix*	4	1	0	0	0	0	0	0	0
	*Ruditapes philippinarum*	23	3	7	0	0	4	0	1	0
	*Solen grandis*	12	5	1	0	0	1	0	2	0
Crab	*Scylla paramamosain*	13	0	1	0	0	0	1	0	0
	*Portunus trituberculatus*	15	0	0	0	0	0	0	0	0
Cephalopods	*Octopus variabilis*	1	0	0	0	0	0	0	0	0
	*Sepiella maindroni*	7	0	3	0	0	0	0	0	0

### Isolation of bacterial strains belonging to *Vibrionaceae*

Bacterial strains belonging to *Vibrionaceae* family were similarly isolated as previously described [9]. Briefly, each sample (10 g) was homogenized in 5 ml of sterile phosphate-buffered saline (PBS, pH 7.4) using a lab blender. The homogenate was diluted 1: 50 into 5 ml Alkaline Peptone Water (APW) (Polypeptone 10 g/l; Sodium chloride 30 g/l; pH8.6) and incubated at 30°C with shaking for 6 h. The APW-enriched culture was diluted 1, 000-fold with PBS, and then 200 μl of the diluted sample were spread on thiosulphate citrate bile salts sucrose (TCBS; Beijing Land Bridge, China) agar plate, and incubated at 37°C for 24 h. Suspected colonies were selected and characterized by the VITEK MS system (bioMerieux, France) with the platform v3.2 according to the manufacturer’s instructions. From the same sample, among clones with the same colony morphology, only one strain was randomly selected. In addition, ethics approval was not requested because no human and experimental animal subjects were involved.

### Polymerase chain reaction (PCR) assay

*V. parahaemolyticus* genomic DNA was extracted similarly as previously study [9]. Briefly, 15 μl glycerol-preserved bacterial strain was inoculated into 5 ml 2.5% Bacto heart infusion (HI; BD Bioscience, USA) broth and incubated at 37°C with shaking for 12 h, followed by centrifugation at 5000 g for 3 min. The genomic DNA was extracted by a QIAamp DNA mini Kit (Qiagen, Germany), and the concentration of DNA solution was measured by a NanoDrop spectrophotometry (ThermoFisher Scientific, USA). PCR was used to detect the presence of the following genetic markers: *tlh*, *tdh*, *trh*, *toxR*, t*oxR/new*, *PGS*, *orf8*, *HU-a*, *ure*, *Mtase*, v*opC* (VPA1321), VPA1376, *vopQ* (VP1680), VP1409, and *vipA2* (VPA1035). PCR amplification was conducted under the following conditions [9]: an initial pre-denaturation step at 95°C for 5 min, succeeded by 30 cycles consisting of denaturation at 94°C for 50 s, annealing at 54°C for 50 s, and extension at 72°C for 50 s. A final extension step was performed at 72°C for 5 min. The PCR products were subsequently visualized using 1% agarose gel electrophoresis. Primers used in this work are listed in Table 2.

**Table 2:** Primers used in this study.

Target	Sequence (forward/reverse, 5ꞌ→3ꞌ)	Size (bp)	Reference
*tlh*	AAAGCGGATTATGCAGAAGCACTG/GCTACTTTCTAGCATTTTCTCTGC	450	[9]
*tdh*	GTAAAGGTCTCTGACTTTTGGAC/TGGAATAGAACCTTCATCTTCACC	269	[9]
*trh*	TTGGCTTCGATATTTTCAGTATCT/CATAACAAACATATGCCCATTTCCG	500	[9]
*toxR*	GTCTTCTGACGCAATCGTTG/ATACGAGTGGTTGCTGTCATG	368	[9]
*toxR*/*new*	TAATGAGGTAGAAACA/ACGTAACGGGCCTACA	651	[9]
*PGS*	TTCGTTTCGCGCCACAACT/TGCGGTGATTATTCGCGTCT	235	[9]
*orf8*	GTTCGCATACAGTTGAGG/AAGTACACAGGAGTGAG	700	[9]
HU-a	CGATAACCTATGAGAAGGGAAACC/CTAGAAGGAAGAATTGATTGTCAAATAATG	474	[9]
*ure*	CTTGTCATCGGGTGTCACTA/GATGTTAGGTTCACCTACTGACT	464	[9]
*Mtase*	GTCTTGTCGAATAGAACTCTGA/TAAGCTCCAAAATCCATACG	683	[9]
*vopC* (VPA1321)	CAGAGTTGGTTTCGCAG/CTGGTACGCCTCTTGGACAG	579	[9]
VPA1376	GCTCTCCTTGGTACCAATCAC/CTGGGATCTTGATGTCAAGGT	1067	[9]
*vopQ* (VP1680)	AAAGATGACCGAGTAATCAGC/TTTCTTAATACGCCTTCGCTA	101	This study
VP1409	TTCTGTGCTCGACTTGTG/TTCAGTGTACTCAACCATCC	106	[63]
*vipA2* (VPA1035)	GTGGGTGTTATGGGCGACTT/AAAGCTCAGTTCAACCGCAA	189	This study

### Crystal violet (CV) staining

The CV staining assay was performed as previously described [25]. Briefly, the overnight bacterial cell culture was diluted 50-fold into 5 ml HI broth and cultured at 37°C with shaking 200 rpm to an OD_600_ value of 1.4. The resultant culture was 50-fold diluted into 2 ml Difco marine (M) broth 2216 (BD Biosciences, USA) in a 96-well plate (Corning Inc., USA) and then grew at 4, 25 or 37°C with shaking at 150 rpm for 24 h. Biofilm cells were stained with 0.1% CV, which was dissolved with 20% acetic acid. The OD_570_ values of acetic acid solutions were measured as the index of CV staining. Biofilms were categorized into three grades based on their OD_570_ values: weak (OD_570_ < 0.2), moderate (0.2 ≤ OD_570_ < 0.7), and strong (OD_570_ ≥ 0.7) [9].

### Motility-associated assays

The swimming and swarming motility were assessed as previously described [26]. Briefly, the overnight cell cultures were diluted 50-fold into 5 ml HI broth and cultured at 37°C with shaking at 200 rpm to an OD_600_ value of 1.4. Thereafter, 2 μl of the culture was inoculated into a semi-solid swim plate (1% Oxoid yeast extract, 1% NaCl [Merck, Germany], and 0.2% Difco Noble agar [BD Biosciences, USA]) or spotted on a swarm plate (1% Oxiod Tryptone, 1% NaCl, 0.5% Oxoid yeast extract, and 1.8% Difco noble agar). Diameter of swimming or swarming zone was measured after incubation at 37°C for 3 or 48 h.

### Antibiotic susceptibility testing (AST)

Antibiotics resistance profiles of *V. parahaemolyticus* isolates were assessed using a VITEK 2 AST-GN09 antimicrobial sensitivity kit (bioMerieux, France) containing ampicillin (AMP), ampicillin/sulbactam (SAM), piperacillin (PIP), piperacillin/tazobactam (TZP), cefazolin (CZ), cefuroxime (CXM), ceftazidime (CAZ), cefepime (FEP), meropenem (MEM), amikacin (AN), gentamicin (CN), ciprofloxacin (CIP), levofloxacin (LEV), and trimethoprim-sulfamethoxazole (SXT). AST was performed as previously described [9]. Briefly, *V. parahaemolyticus* cells were introduced into a test tube containing 3 ml of a 0.45% NaCl solution. The turbidity of the resulting bacterial solution was adjusted to match that of the 0.5 McFarland standard using a DensiCHEK Plus turbidimeter from bioMerieux, France. Subsequently, 145 μl bacterial suspension was taken in a separate test tube. The AST for *V. parahaemolyticus* isolates was conducted by determining the minimum inhibitory concentrations (MICs). This was achieved using a VITEK2 Compact automatic microbial analyzer, a device manufactured by bioMérieux in France. The results were categorized as resistant (R), intermediate (I), and susceptible (S).

### Replicates and statistical methods

PCR and AST were performed three times with the similar results. The CV staining assay, swimming and swarming motility were performed at least three independent bacterial cultures with three replicates for each, and the results were expressed as the mean ± standard deviation (SD). Paired Student’s *t*-tests and two-way ANOVA with Tukey’s post hoc corrections for multiple comparisons were used to calculate statistical significance, with *P* < 0.01 considered significant.

## Results

### Prevalence of *Vibrionaceae* family in retail seafood products

Colonies suspected to be *Vibrionaceae* family were randomly selected from 30 types of seafood on selective TCBS agar plates for further identification. A total of 240 colonies were identified, with the breakdown as follows: 146 *Photobacterium damselae* (*P*. *damselae*), 59 *V. parahaemolyticus*, 18 *V. campbellii*, 11 *V. alginolyticus*, 3 *V. vulnificus*, 1 *V. mimicus*, 1 *V. fluvialis*, and 1 *V. harveyi* (Table 3). Among the seafood products tested, 50.0% (15 types) were positive for *V. parahaemolyticus*, affecting 9 fish species, 3 shrimp species, and 3 shellfish species. *P*. *damselae* was detected in 63.3% (19 types), including 11 fish species, 4 shrimp species, 2 shellfish species, 1 crab species, and 1 cephalopods species. *V. campbellii* was found in 30.0% (9 types) of the seafood products, affecting 5 fish species, 3 shrimp species, and 1 crab species (Table 1). *V. alginolyticus* was isolated in 26.7% (8 types) of the seafood products, affecting 6 fish species, and 2 shellfish species (Table 1). The overall detection rate for *V. parahaemolyticus* in seafood was 8.2%, with rates of 7.9% in fish, 7.5% in shrimp, and 23.1% in shellfish. For *P*. *damselae*, the detection rate was higher at 20.3%, with 18.5% in fish, 29.9% in shrimp, 20.5% in shellfish, 3.6% in crabs, and 37.5% in cephalopods (Table 3). Other *Vibrionaceae* family had very low detection rates, with only *V. campbellii* exceeding 2.0%.

**Table 3:** Isolates and detection rates of Vibrio species from seafood samples.

Species	Number of samples	Number of isolates (detection rate, %)							
		*V. parahaemolyticus*	*Photobacterium damselae*	*V. mimicus*	*V. fluvialis*	*V. alginolyticus*	*V. campbellii*	*V. vulnificus*	*V. harveyi*
Fish	509	40 (7.9)	94 (18.5)	1 (0.2)	0 (0.0)	6 (1.2)	13 (2.6)	0 (0.0)	0 (0.0)
Shrimp	134	10 (7.5)	40 (29.9)	0 (0.0)	1 (0.7)	0 (0.0)	4 (3.0)	0 (0.0)	1 (0.7)
Shellfish	39	9 (23.1)	8 (20.5)	0 (0.0)	0 (0.0)	5 (12.8)	0 (0.0)	3 (7.7)	0 (0.0)
Crab	28	0 (0.0)	1 (3.6)	0 (0.0)	0 (0.0)	0 (0.0)	1 (3.6)	0 (0.0)	0 (0.0)
Cephalopods	8	0 (0.0)	3 (37.5)	0 (0.0)	0 (0.0)	0 (0.0)	0 (0.0)	0 (0.0)	0 (0.0)
Total	718	59 (8.2)	146 (20.3)	1 (0.1)	1 (0.1)	11 (1.5)	18 (2.5)	3 (0.4)	1 (0.1)

### Virulence gene profiles in *V. parahaemolyticus* isolates

*V. parahaemolyticus* is the leading cause of seafood-associated gastroenteritis in coastal areas [27, 28]. Therefore, in this study, the virulence gene profiles in the 59 *V. parahaemolyticus* isolates were examined. As detailed in Table 4, all isolates contained the gene *tlh* and *toxR*, but none were positive for *tdh* or *trh*. Furthermore, none of the isolates carried the genes *toxR*/*new*, HU-α, *ure*, *Mtase*, *vopC* and VPA1376, with the exception of one isolate that possessed the *orf8* gene. Additionally, 45.8% (27/59) of the isolates carried the *PGS* sequence, 98.3% (58/59) carried *vopQ*, 39.0% (23/59) carried VP1409, and 100% (59/59) carried *vipA2*. In summary, these findings suggest that the majority of the isolates were non-pathogenic, although the presence of certain virulence genes indicates that a small proportion may still have pathogenic potential.

**Table 4:** Presence of virulence genes in *V. parahaemolyticus* isolates.

Strains	*tlh*	*tdh*	*trh*	*toxR*	*toxR*/new	*PGS*	*orf8*	HU-α	*ure*	*MTase*	*vopC*	VPA1376	*vopQ*	VP1409	*vipA2*
VP_QD_1	+	-	-	+	**-**	-	-	-	-	-	-	-	+	-	+
VP_QD_2	+	-	-	+	-	-	-	-	-	-	-	-	+	-	+
VP_QD_3	+	-	-	+	-	+	-	-	-	-	-	-	+	+	+
VP_QD_4	+	-	-	+	-	+	-	-	-	-	-	-	+	-	+
VP_QD_5	+	-	-	+	-	-	-	-	-	-	-	-	+	-	+
VP_QD_6	+	-	-	+	-	-	-	-	-	-	-	-	+	+	+
VP_QD_7	+	-	-	+	-	-	-	-	-	-	-	-	+	+	+
VP_QD_8	+	-	-	+	-	+	-	-	-	-	-	-	+	+	+
VP_QD_9	+	-	-	+	-	-	-	-	-	-	-	-	+	-	+
VP_QD_10	+	-	-	+	-	-	-	-	-	-	-	-	+	+	+
VP_QD_11	+	-	-	+	-	+	-	-	-	-	-	-	+	-	+
VP_QD_12	+	-	-	+	-	+	-	-	-	-	-	-	+	+	+
VP_QD_13	+	-	-	+	-	-	-	-	-	-	-	-	+	+	+
VP_QD_14	+	-	-	+	-	+	-	-	-	-	-	-	+	-	+
VP_QD_15	+	-	-	+	-	-	-	-	-	-	-	-	+	-	+
VP_QD_16	+	-	-	+	-	-	-	-	-	-	-	-	+	-	+
VP_QD_17	+	-	-	+	-	+	-	-	-	-	-	-	+	+	+
VP_QD_18	+	-	-	+	-	-	-	-	-	-	-	-	+	-	+
VP_QD_19	+	-	-	+	-	+	-	-	-	-	-	-	+	+	+
VP_QD_20	+	-	-	+	-	-	-	-	-	-	-	-	+	-	+
VP_QD_21	+	-	-	+	-	-	-	-	-	-	-	-	+	+	+
VP_QD_22	+	-	-	+	-	-	-	-	-	-	-	-	+	-	+
VP_QD_23	+	-	-	+	-	+	-	-	-	-	-	-	+	-	+
VP_QD_24	+	-	-	+	-	+	-	-	-	-	-	-	+	-	+
VP_QD_25	+	-	-	+	-	-	-	-	-	-	-	-	+	+	+
VP_QD_26	+	-	-	+	-	-	-	-	-	-	-	-	+	+	+
VP_QD_27	+	-	-	+	-	+	-	-	-	-	-	-	+	+	+
VP_QD_28	+	-	-	+	-	-	-	-	-	-	-	-	+	+	+
VP_QD_29	+	-	-	+	-	+	-	-	-	-	-	-	+	-	+
VP_QD_30	+	-	-	+	-	+	-	-	-	-	-	-	+	+	+
VP_QD_31	+	-	-	+	-	-	-	-	-	-	-	-	+	-	+
VP_QD_32	+	-	-	+	-	-	-	-	-	-	-	-	+	-	+
VP_QD_33	+	-	-	+	-	-	-	-	-	-	-	-	+	-	+
VP_QD_34	+	-	-	+	-	-	-	-	-	-	-	-	+	-	+
VP_QD_35	+	-	-	+	-	-	-	-	-	-	-	-	+	-	+
VP_QD_36	+	-	-	+	-	-	-	-	-	-	-	-	+	-	+
VP_QD_37	+	-	-	+	-	-	-	-	-	-	-	-	+	-	+
VP_QD_38	+	-	-	+	-	-	-	-	-	-	-	-	+	-	+
VP_QD_39	+	-	-	+	-	-	-	-	-	-	-	-	+	-	+
VP_QD_40	+	-	-	+	-	+	-	-	-	-	-	-	+	-	+
VP_QD_41	+	-	-	+	-	-	-	-	-	-	-	-	+	+	+
VP_QD_42	+	-	-	+	-	-	-	-	-	-	-	-	+	-	+
VP_QD_43	+	-	-	+	-	+	-	-	-	-	-	-	+	+	+
VP_QD_44	+	-	-	+	-	+	+	-	-	-	-	-	+	+	+
VP_QD_45	+	-	-	+	-	+	-	-	-	-	-	-	+	+	+
VP_QD_46	+	-	-	+	-	+	-	-	-	-	-	-	+	-	+
VP_QD_47	+	-	-	+	-	-	-	-	-	-	-	-	+	-	+
VP_QD_48	+	-	-	+	-	+	-	-	-	-	-	-	+	+	+
VP_QD_49	+	-	-	+	-	+	-	-	-	-	-	-	+	-	+
VP_QD_50	+	-	-	+	-	+	-	-	-	-	-	-	+	-	+
VP_QD_51	+	-	-	+	-	+	-	-	-	-	-	-	+	+	+
VP_QD_52	+	-	-	+	-	+	-	-	-	-	-	-	+	-	+
VP_QD_53	+	-	-	+	-	-	-	-	-	-	-	-	+	-	+
VP_QD_54	+	-	-	+	-	-	-	-	-	-	-	-	+	-	+
VP_QD_55	+	-	-	+	-	+	-	-	-	-	-	-	+	+	+
VP_QD_56	+	-	-	+	-	+	-	-	-	-	-	-	+	+	+
VP_QD_57	+	-	-	+	-	+	-	-	-	-	-	-	+	-	+
VP_QD_58	+	-	-	+	-	-	-	-	-	-	-	-	-	-	+
VP_QD_59	+	-	-	+	-	+	-	-	-	-	-	-	+	-	+

### Biofilm formation by the *V. parahaemolyticus* isolates

**Fig 1:**
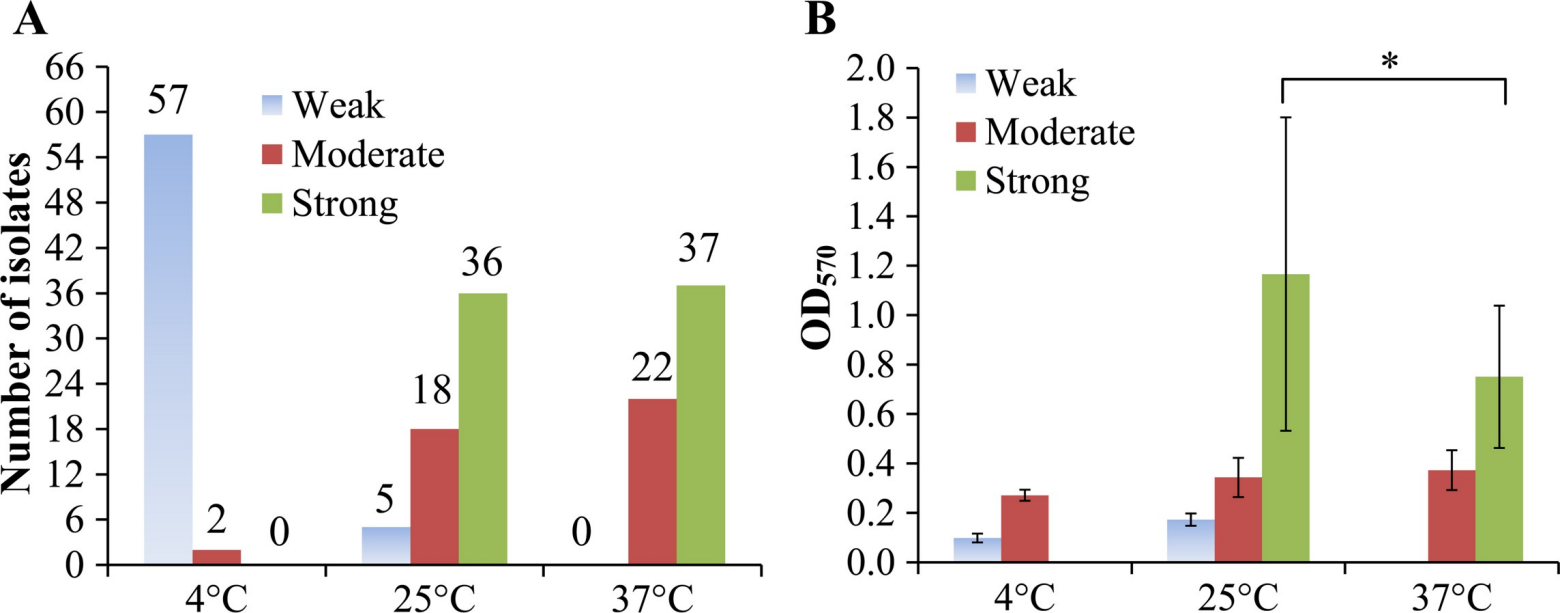
Biofilm formation by the *V. parahaemolyticus* isolates. A) Statistics on the number of biofilm producers. The number at the top of each bar represents the count of biofilm producers. B) The results of crystal violet staining assays. The asterisk (*) indicates a statistically significant difference (*P* < 0.01).

The CV staining assay was employed to assess the biofilm-forming capacities of the 59 *V. parahaemolyticus* isolates. As depicted in Fig 1A, all isolates demonstrated the ability to form biofilms. At 4°C, the majority were classified as weak biofilm producers, with only two isolates exhibiting moderate biofilm formation (Fig 1A and 1B). In contrast, at 25°C and 37°C, the majority of isolates were identified as moderate or strong biofilm producers, with a notable absence of weak biofilm formers (Fig 1A and 1B). Furthermore, the strong biofilm-forming isolates displayed a significantly higher capacity for biofilm formation at 25°C compared to 37°C (Fig 1B).

### Swimming and swarming motility of the *V. parahaemolyticus* isolates

**Fig 2:**
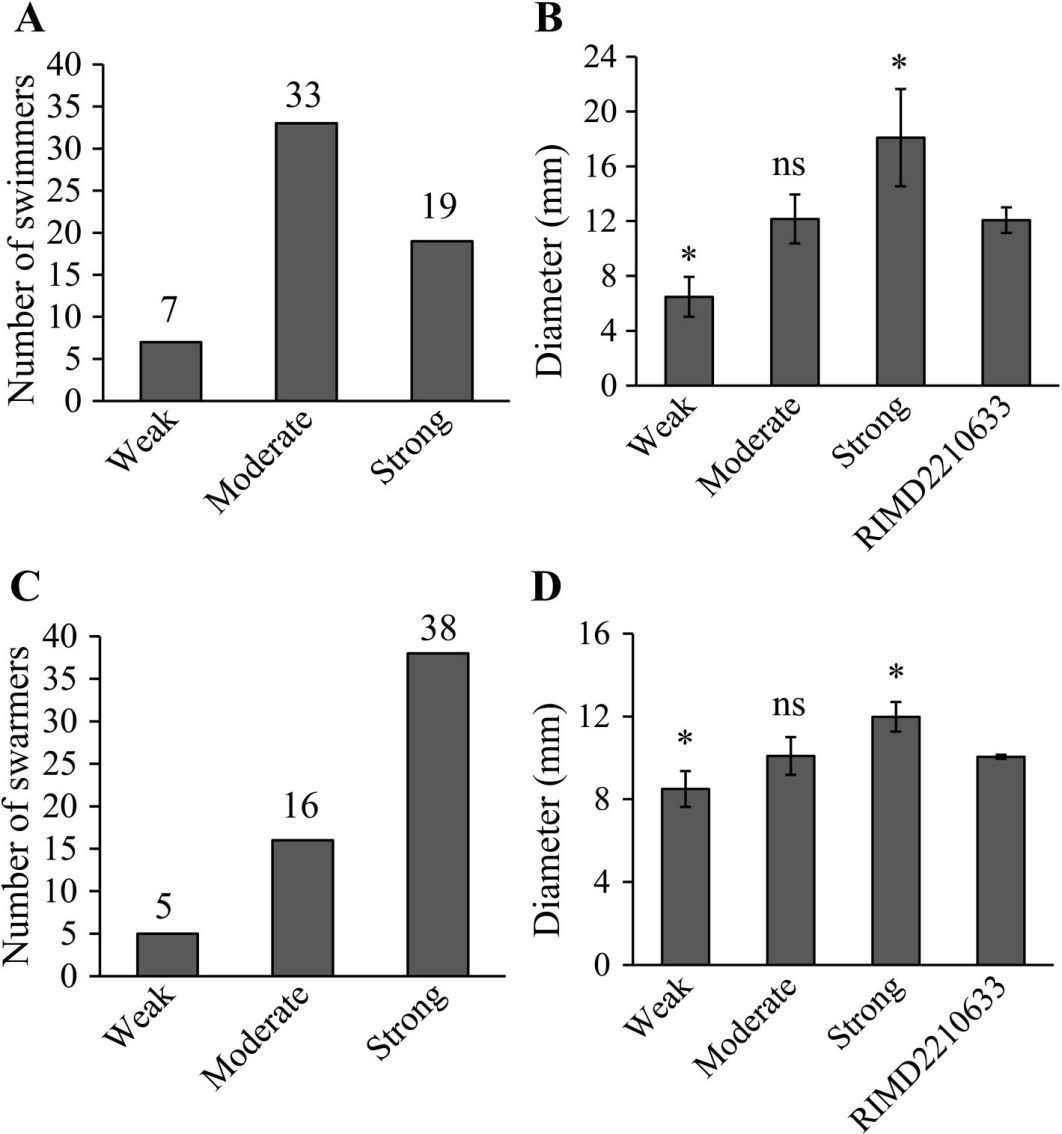
The swimming and swarming motility of the *V. parahaemolyticus* isolates. Statistics on the number of swimmers (A) and swarmers (C) and the results of swimming (B) and swarming motility (D). The number at the top of each bar indicates the count of swimmers or swarmers. The asterisks (*) indicate significant differences compared to *V. parahaemolyticus* RIMD2210633 (*P* < 0.01). The ‘ns’ symbols represent no significant differences (*P* > 0.01).

*V. parahaemolyticus* is capable of swimming in liquids and swarming over surfaces or in viscous conditions [29]. In this study, we investigated the swimming and swarming motility of each isolate, assessing the levels of these two types of motility using a previously described method [9]. As shown in Fig 2A and 2B, all isolates exhibited swimming capabilities, with 7 showing weak, 33 moderate, and 19 strong swimming abilities. Likewise, all isolates displayed swarming anility, with 5 showing weak, 16 moderate, and 38 strong swarming abilities (Fig 2C and 2D). These findings align with previous observations that the majority of V. *parahaemolyticus* isolates possess notably strong motility [9, 30].

### Antibiotics resistance profiles of the *V. parahaemolyticus* isolates

AST was performed on 59 *V. parahaemolyticus* isolates using a panel of 14 antibiotics. As shown in Table 5, sensitivity to ampicillin was observed in only 8.5% of the isolates, with a significant majority (83.0%) exhibiting resistance. A high level of intermediate resistance was noted for cefuroxime (93.2%) and cefazolin (84.7%). Conversely, all isolates displayed sensitivity to the remaining 11 antibiotics listed in Table 5.

**Table 5:** Antibiotics resistance profiles of *V. parahaemolyticus* isolates.

Antibiotics	Number (%) of S	Number (%) of I	Number (%) of R
Ampicillin	5 (8.5)	5 (8.5)	49 (83.0)
Ampicillin/sulbactam	59 (100.0)	0 (0.0)	0 (0.0)
Piperacillin	59 (100.0)	0 (0.0)	0 (0.0)
Piperacillin/tazobactam	59 (100.0)	0 (0.0)	0 (0.0)
Cefazolin	9(15.3)	50 (84.7)	0 (0.0)
Cefuroxime	4 (6.8)	55(93.2)	0 (0.0)
Ceftazidime	59 (100.0)	0 (0.0)	0 (0.0)
Cefepime	59 (100.0)	0 (0.0)	0 (0.0)
Meropenem	59 (100.0)	0 (0.0)	0 (0.0)
Amikacin	59 (100.0)	0 (0.0)	0 (0.0)
Gentamicin	59 (100.0)	0 (0.0)	0 (0.0)
Ciprofloxacin	59 (100.0)	0 (0.0)	0 (0.0)
Levofloxacin	59 (100.0)	0 (0.0)	0 (0.0)
Trimethoprim-sulfamethoxazole	59 (100.0)	0 (0.0)	0 (0.0)

## Discussion

In this study, a total of 240 bacterial isolates belonging to *Vibrionaceae* family were recovered from 718 seafood samples collected from the retail seafood products market in Qidong city from June to November of 2023. Of these, 146 were identified as *P*. *damselae*, 59 as *V. parahaemolyticus*, 18 as *V. campbellii*, 11 as *V. alginolyticus*, and 6 as other *Vibrio* species (Table 3). The isolation rates were 20.3% for *P*. *damselae*, 8.2% for *V. parahaemolyticus*, 2.5% for *V. campbellii*, and less than 2.0% for the remaining *Vibrionaceae* family (Table 3). *P*. *damselae*, a significant pathogen in marine ecosystems, is known to cause infections in various marine animals such as fish, molluscs, crustaceans, and even cetaceans [31]. It poses a risk to human health as an opportunistic pathogen, potentially leading to fatal outcomes through the consumption of raw seafood or exposure to seawater [31]. Therefore, it is imperative that the safety concerns associated with *P*. *damsela*e in seafood are addressed with due diligence. Additionally, extensive literature exists that examines the prevalence of *V. parahaemolyticus* in seafood products [20, 21, 27, 28, 32–35]; however, the detection rates reported vary significantly across studies. For instance, studies have reported detection rates of 28.8% in seafood from ‘‘La Nueva Viga” market in Mexico City [36], 40.3% from 7 stations along the Korean coast [37], 28.57% in the Greater Sacramento area in California [34], and 21.7% in local seafood market of Northern Thailand [33]. While the isolation rate of *V. parahaemolyticus* observed in our study (8.2%) was significantly lower than rates reported in some existing literature, the underlying reasons for this discrepancy remain unclear.

*V. parahaemolyticus* is the primary causative agent of seafood-associated food poisoning. The presence of *tdh* and *trh* genes serves as molecular indicators of pathogenicity in *V. parahaemolyticus* isolates [5]. However, the majority of *V. parahaemolyticus* found in the environment are non-pathogenic and typically lack the *tdh* and/or *trh* genes [30, 35, 38, 39]. It is noteworthy that all *V. parahaemolyticus* strains examined in this study were devoid of both *tdh* and *trh* (Table 4). Despite this, there are studies suggesting that *tdh*/trh-negative strains can still cause human infection [9, 40]. This implies that even these ‘non-pathogenic’ strains should be considered in the realm of seafood safety monitoring. Several DNA markers, such as *toxRS*/*new*, *orf8*, HU-α, and *PGS* sequence, have been utilized to differentiate strains that belong to the ‘pandemic group’ [10–13]. The findings of this study indicate that 45.8% of the isolates contained the *PGS* sequence (Table 4), which suggests that this marker may not be reliable for identifying the ‘pandemic group’, considering all strains in this study were non-pathogenic. Furthermore, none of the isolates were found to carry *vopC* and VPA1376, which are located within the Vp-PAI gene cluster (T3SS2), while 98.3% of the isolates were positive for *vopQ*, located in the T3SS1 gene cluster (Table 4). Additionally, all isolates were found to carry *vopA2*, which is part of the T6SS2 gene cluster, whereas only 39.0% of the isolates contained VP1409, part of the T6SS1 gene cluster (Table 4). These results align with the understanding that the T3SS1 and T6SS2 gene clusters are ubiquitous in all *V. parahaemolyticus* isolates, while the T3SS2 and T6SS1 gene clusters are more commonly identified in clinical isolates [41–43].

All 59 isolates of *V. parahaemolyticus* exhibited swimming and swarming motility, as depicted in Fig 2. These motilities are driven by polar and lateral flagella, respectively, and are crucial for the biofilm formation, especially in the initial stage, of *V. parahaemolyticus* [44]. Notably, all isolates were capable of forming biofilms, with the majority being weak producers at 4°C and moderate to strong producers at 25°C or 37°C (Fig 1). The capacity for biofilm formation at lower temperatures, such as 4°C, may be significant for the bacteria’s ability to persist on seafood surfaces over extended periods [18]. The effect of culture temperature on *V. parahaemolyticus* biofilm formation was observed, with a markedly higher biofilm-forming ability at 25°C compared to 15°C and 37°C [45]. However, another study identified 37°C as the optimal temperature for biofilm formation by this bacterium [46]. The data presented here indicate that while the number of strong biofilm producers was similar at both at 25°C and 37°C, the biofilms produced at 25°C were significantly stronger than those at 37°C (Fig 1B). These results indicate that variables such as strain differences, culture media, and the type of culture containers can significantly influence the outcomes of biofilm-related phenotypes.

Bacterial biofilms pose significant challenges to food safety in the food industry [47]. Conventional approaches are insufficient to completely remove biofilms formed by *Vibrionaceae* family. However, advancements have been made with the development of safe and effective anti-biofilm strategies [48–50]. For instance, natural compounds such as eugenol [51], carvacrol [52], quercetin [53], as well as essential oils [54, 55], have demonstrated the ability to inhibit biofilm formation. Additionally, physical treatments like ultraviolet [56] and enzymatic treatments with flavourzyme [57] have shown promise in controlling biofilms in food products. Furthermore, the use of bacteriophages [58], lactic acid bacteria [59], and the manipulation of environmental conditions, such as altering glucose concentration [60, 61], have been found to impact the growth of foodborne pathogenic biofilms on food surfaces. These approaches offer potential strategies for combating pathogenic microorganisms in the seafood industry, providing a means to enhance food safety.

The majority of the *V. parahaemolyticus* strains examined in this study exhibited high resistance to ampicillin, while displaying intermediate resistance to cefuroxime and cefazolin (Table 5). In contrast, clinical isolates from the stool samples of diarrhea patients in Nantong during 2018–2020 showed 68.2% and 75.6% high and moderate resistance to cefuroxime and cefazolin, respectively [9]. This may indicate a significant divergence in the antibiotic resistance patterns between pathogenic and non-pathogenic *V. parahaemolyticus* strains in Nantong city. The common resistance to ampicillin among *V. parahaemolyticus* isolates is attributed to the presence of *bla*_CARB-17_ in all tested strains. This gene encodes a novel member of the CARB-17 family of β-lactamases, which confers intrinsic resistance to penicillins [62]. Furthermore, *V. parahaemolyticus* strains isolated from ready-to-eat foods in China have been found to carry class 1 integrons with genetic structures such as *dfrA14*-*bla*_VEB-1_-*aadB* and *bla*_VEB-1_-*aadB*-*arr2*-*cmlA*-*bla*_OXA-10_-*aadA1*, which are associated with resistance to a range of antibiotics, including ampicillin [17]. Nevertheless, additional research is warranted to explore the prevalence of antibiotic resistance genes among the *V. parahaemolyticus* strains identified in this study. This will provide a more comprehensive understanding of the resistance mechanisms in *V. parahaemolyticus*.

In summary, this study conducted an assessment of *Vibrionaceae* family in retail seafood products available in the Qidong market during the summer of 2023. The findings revealed that *P*. *damselae* and *V. parahaemolyticus* were the predominant species, with prevalence rates of 20.3% and 8.2%, respectively. Among the 59 *V. parahaemolyticus* strains isolated, none were pathogenic. These *V. parahaemolyticus* strains demonstrated proficiency in swimming and swarming behaviors, and a notable capacity to form biofilms. Furthermore, the study identified that the majority of *V. parahaemolyticus* isolates exhibited high resistance to ampicillin, intermediate resistance to cefuroxime and cefazolin, and sensitivity to other antibiotics tested. The data presented here would provide theoretical guidance for food safety, and may be beneficial for controlling and treating seafood-related illnesses caused by *Vibrionaceae* family in Qidong City.
